# A composite biomarker of neutrophil-lymphocyte ratio and hemoglobin level correlates with clinical response to PD-1 and PD-L1 inhibitors in advanced non-small cell lung cancers

**DOI:** 10.1186/s12885-021-08194-9

**Published:** 2021-04-21

**Authors:** Kristin L. Ayers, Meng Ma, Gaspard Debussche, David Corrigan, Jonathan McCafferty, Kyeryoung Lee, Scott Newman, Xiang Zhou, Fred R. Hirsch, Philip C. Mack, Jane J. Liu, Eric E. Schadt, Rong Chen, Shuyu D. Li

**Affiliations:** 1 Sema4, a Mount Sinai Venture, 333 Ludlow Street, Stamford, CT 06902 USA; 2 Center of Thoracic Oncology/Tisch Cancer Institute and Icahn School of Medicine, Mount Sinai, 1 Gustave L. Levy Pl, New York, NY 10029 USA; 3 Illinois Cancer Care, 8940 N Wood Sage Rd, Peoria, IL 61615 USA

**Keywords:** Non-small cell lung cancer, Immune checkpoint inhibitor, Neutrophil-lymphocyte ratio, Anemia, Biomarker

## Abstract

**Background:**

Immune checkpoint inhibitors (ICIs) have been incorporated into various clinical oncology guidelines for systemic treatment of advanced non-small cell lung cancers (aNSCLC). However, less than 50% (and 20%) of the patients responded to the therapy as a first (or second) line of therapy. PD-L1 immunohistochemistry (IHC) is an extensively studied biomarker of response to ICI, but results from this test have equivocal predictive power. In order to identify other biomarkers that support clinical decision-making around whether to treat with ICIs or not, we performed a retrospective study of patients with aNSCLC who underwent ICI-based therapy in the Mount Sinai Health System between 2014 and 2019.

**Methods:**

We analyzed data from standard laboratory tests performed in patients as a part of the routine clinical workup during treatment, including complete blood counts (CBC) and a comprehensive metabolic panel (CMP), to correlate test results with clinical response and survival.

**Results:**

Of 11,138 NSCLC patients identified, 249 had been treated with ICIs. We found associations between high neutrophil-to-lymphocyte ratio (NLR ≥ 5) and poor survival in ICI-treated NSCLC. We further observed that sustained high NLR after initiation of treatment had a more profound impact on survival than baseline NLR, regardless of PD-L1 status. Hazard ratios when comparing patients with NLR ≥ 5 vs. NLR < 5 are 1.7 (*p* = 0.02), 3.4 (*p* = 4.2 × 10^− 8^), and 3.9 (*p* = 1.4 × 10^− 6^) at baseline, 2–8 weeks, and 8–14 weeks after treatment start, respectively. Mild anemia, defined as hemoglobin (HGB) less than 12 g/dL was correlated with survival independently of NLR. Finally, we developed a composite NLR and HGB biomarker. Patients with pretreatment NLR ≥ 5 and HGB < 12 g/dL had a median overall survival (OS) of 8.0 months (95% CI 4.5–11.5) compared to the rest of the cohort with a median OS not reached (95% CI 15.9-NE, *p* = 1.8 × 10^− 5^), and a hazard ratio of 2.6 (95% CI 1.7–4.1, *p* = 3.5 × 10^− 5^).

**Conclusions:**

We developed a novel composite biomarker for ICI-based therapy in NSCLC based on routine CBC tests, which may provide meaningful clinical utility to guide treatment decision. The results suggest that treatment of anemia to elevate HGB before initiation of ICI therapy may improve patient outcomes or the use of alternative non-chemotherapy containing regimens.

**Supplementary Information:**

The online version contains supplementary material available at 10.1186/s12885-021-08194-9.

## Background

Regimens containing immune checkpoint inhibitors (ICIs) such as anti-PD1 or anti-PD-L1 antibodies have been the standard-of-care therapy for the treatment of advanced NSCLC (aNSCLC) without identifiable molecular driver mutations. However, even in the first-line setting, less than 50% of the patients respond to this type of therapy [1–3], and the response rates are less than 20% when used as part of 2nd line treatments [4–6]. Subsequently, significant efforts have been made to identify biomarkers that are predictive of treatment response. Although PD-L1 has been widely used as a patient stratification biomarker for making treatment decisions, its predictive power is less than optimal as the response rate in PDL1 positive and negative individuals only modestly differs [1, 2].

In order to be effective, immunotherapies must encourage a robust innate and/or adaptive immune response towards the patient’s tumor. It is, therefore, reasonable to ask if populations of immune effector cells such as neutrophils and lymphocytes shift over the course of immunotherapy treatment, and if baseline or in-treatment levels of these cells affect ICI response. For example, the neutrophil-to-lymphocyte ratio (NLR) is a recognized prognostic marker. A high NLR at baseline or during treatment correlates with poor prognosis such as shorter overall survival (OS), shorter progression-free survival (PFS), or lack of response to therapy in lung, colorectal, kidney and many other solid cancers [7]. Recently, blood counts have been heavily studied in relation to melanoma immunotherapy response [8], and several studies have also explored their utility in the context of NSCLC (Supplementary Table 1) [9–23]. These previous studies have generally focused on pretreatment counts rather than changes over the course of therapy, or have been relatively small when examining post treatment effects with less than 160 individuals being studied. Further, most existing studies have not considered the overall status of the patient, other than the ECOG score or site specific metastases.

In addition to blood counts, many other lab tests assess electrolyte imbalances, kidney function, and liver function. These frequently repeated tests monitor the status of the patient’s health during the course of cancer treatment and progression. Previous studies have shown associations between cancer outcomes and standard lab tests. For example, low baseline serum sodium concentration has been associated with shorter OS in a cohort of 197 NSCLC patients on immunotherapy [24]. Low pretreatment serum albumin has been associated with shorter PFS and early progression [25]. Pretreatment lactate dehydrogenase (LDH) levels greater than the upper limit of reference range was associated with shorter OS in a cohort of 161 individuals [26], and in a meta-analysis (*n* = 1136), higher pretreatment LDH levels were correlated with significant shorter PFS and OS for ICI therapy in aNSCLC [27]. Anemia has been associated with poor survival in cancers in general, and more than 30% of lung cancer patients experience anemia, and its incidence after chemotherapy has been estimated at 80% [28].

Electronic medical records provide a valuable resource for retrospective analysis of real-world patient data for biomarker discovery and validation. Typically, these real-world patient populations, although extremely large, span many years and, reflecting the evolution of treatment guidelines, are heterogeneous in terms of therapies received and laboratory tests performed. However, in spite of this longitudinal heterogeneity, most cancer patients receive a standard battery of laboratory tests including a comprehensive metabolic panel (CMP) and complete blood counts (CBC) throughout their course of therapy. When analyzed within a large enough cohort, it is possible that these standard tests have additional utility and potential prognostic or predictive power beyond their original intent.

In this study we analyzed the medical records of 11,138 NSCLC patients in the Mount Sinai Health System electronic health record (EHR) database, 249 of whom were treated with the PD-L1/PD-1 ICIs nivolumab, atezolizumab, or pembrolizumab for metastatic disease at any line of therapy. We first tested if we could reproduce previously reported correlations between NLR and clinical outcomes in ICI-treated aNSCLC from our data. We also evaluated additional factors that can influence neutrophil levels such as the timing of chemotherapy over a patient’s cancer journey, infections, administration of white blood cell growth factors (which often occur with chemotherapy treatments), and overall patient health to determine if these effects impact the association of NLR and ICI treatment outcomes. We further analyzed common, readily available lab test results such as CBC and CMP to identify markers that correlate with response and survival independently of NLR. Finally, we developed a novel composite biomarker to better predict ICI treatment response and clinical outcomes in aNSCLC.

## Methods

### Study population and data collection

We identified 11,138 NSCLC patients in the Mount Sinai Health System (MSHS) EHR based on pathology confirmed diagnosis. From this set of patients we identified 249 patients that had received nivolumab, pembrolizumab or atezolizumab at any line of therapy prior to December 15, 2018 and were followed until May 2019. Response was defined by radiographic response extracted from the clinical notes, and not according to the Response Evaluation Criteria In Solid Tumors guidelines for immune-based therapeutics (iRECIST), since formal assessment based on iRECIST is not typically performed in routine clinical practice. Response was categorized as complete or partial response (CR/PR), stable disease (SD), progressive disease (PD) and putative hyper-progressive disease (HPD). We determined HPD, a sudden acceleration of tumor growth, upon anti-PD1/PD-L1 treatment based on time-to-treatment failure (TTF) < 2 months, an approximate definition used in previous clinical studies [29, 30], specifically when one of the following conditions are met: (1) patients died within 60 days after the initiation of treatment and the death was not due to immune-related adverse events (irAE); (2) patients had documented disease progression within 60 days after the initiation of treatment.

We queried electronic health records to obtain sex, smoking status, tumor histology, epidermal growth factor-receptor (EGFR) and anaplastic lymphoma kinase (ALK) mutation status, IHC PD-L1 testing status, previous or concurrent chemotherapy, concurrent infection, tumor response status when available, first treatment date, last treatment date, and date of death or last follow-up date. Time-to-treatment discontinuation (TTD) was computed as: “the last administration date” minus “the first administration date” plus 1 day. The treatment discontinuation event was defined as permanent discontinuation of treatment [31]. Permanent discontinuation was determined when one of the following conditions was met: (1) having a subsequent line of systemic therapy after the anti-PD1/PD-L1-containing regimen; (2) having a date of death while on the anti-PD1/PD-L1-containing regimen; or (3) having a gap of more than 120 days between the last administration of anti-PD1/PD-L1 therapy and the patient’s last visit, if no other systemic therapy could be identified after the anti-PD1/PD-L1 treatment. Patients without permanent discontinuation were censored at their last administration date of the anti-PD1/PD-L1 therapy. OS was computed as the time from the first administration date of the anti-PD1/PD-L1 therapy to the death date recorded in the MSHS death registry. The OS event is defined as death. Patients without an event were censored at their last visit date.

### Blood counts and other lab tests

For the 249 aNSCLC patients, we extracted results of CBC tests for lymphocytes, monocytes, neutrophils, eosinophils, basophils, white blood cells, red blood cells and platelets. Baseline values were computed at the date nearest to or on the first treatment date within 60 days prior, and up to 97% of patients have a value within the 2 weeks prior treatment start. Lab values occurring in time ranges (e.g. 2–8 weeks) were computed as the mean of all lab values recorded within that time frame for an individual. All tests from the CBC panel and the CMP that had less than 3% missing data at baseline and less than 10% missing data at the 2–8 weeks interval were included in the analyses. Normal lab value ranges were taken from https://cllsociety.org/toolbox/normal-lab-values/ as well as previous studies for aNSCLC (see Supplementary Table 2 reference ranges and the list of lab tests examined).

### Statistical methods

Hazard ratios were computed for individuals outside the reference range versus those in the normal range using cox proportional hazards (only unidirectional thresholds were considered and thus individuals still may be outside the reference range for the normal group). Wald test 95% confidence intervals (CIs) and *p*-values were computed. Odds ratio (OR) were computed with Fisher’s test for the NLR contingency tables or with logistic regression for the individual lab tests using two-sided tests. Neutrophils were analyzed using a linear model and effect sizes (betas) were reported. KM curves with multiple comparison groups report the log-rank *p*-values for the overall model. Reported *p* values were not adjusted for multiple testing. The Bonferroni corrected p-value for the exploratory analysis of all lab tests for a given time point and outcome would be 0.0015 (0.05/33). However, because many of these lab tests are highly correlated, this value may be overly conservative.

## Results

### Study population and the analyzed datasets

**Fig. 1:**
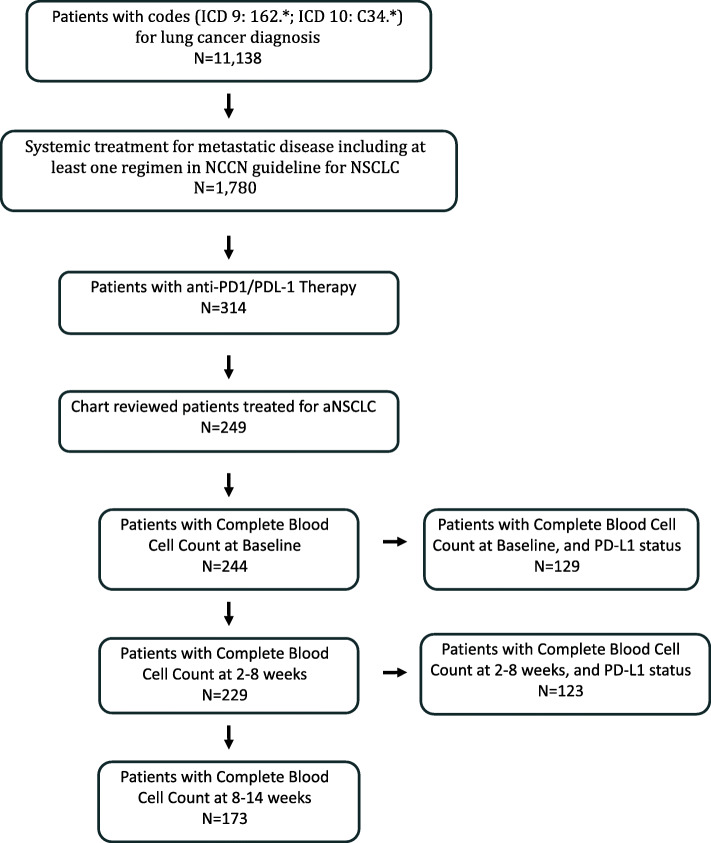
A flow chart of patient population with available data analyzed in the study

Figure 1 summarizes the study population and the number of patients with available complete blood count data at various time points. A subset of the population also had available PD-L1 status for analysis.

### NLR correlates with clinical outcomes in ICI-treated aNSCLC

**Fig. 2:**
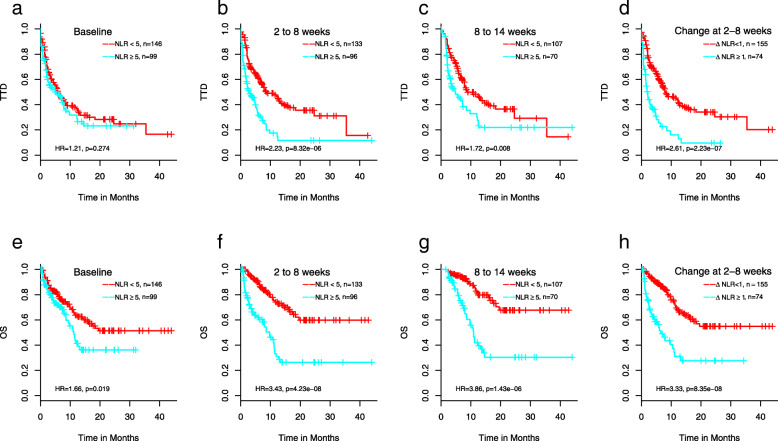
Association of NLR with TTD (**a**-**d**) and OS (**e**-**h**). Kaplan-Meier curves are shown for NLR ≥ 5 and NLR < 5 patients at baseline (time 0, < 30 days prior to treatment) (**a**, **e**), 2–8 weeks (**b**, **f**), and 8–14 weeks (**c**, **g**), or increase in NLR from baseline to 2–8 weeks ≥1 and < 1 (D, H)

Figure 2 demonstrates that higher NLR is correlated with shorter TTD (time-to treatment discontinuation) (Fig. 2, A-C) and OS (Fig. 2, E-G) at all 3 time points except for TTD at baseline. The hazard ratios (HRs) with 95% confidence intervals (CI) for TTD and OS, respectively, at baseline are 1.2 (*p* = 0.27, CI = 0.86–1.72) and 1.7 (*p* = 0.02, CI = 1.1–2.5), at 2–8 weeks are 2.2 (*p* = 8.3 × 10^− 6^, CI = 1.5–3.2) and 3.4 (*p* = 4.2 × 10^− 8^, CI = 2.2–5.3), and at 8–14 weeks are 1.7 (*p* = 0.008, CI = 1.2–2.6) and 3.9 (*p* = 1.4 × 10^− 6^, CI = 2.2–6.7). Increasing NLR between baseline and 2–8 weeks (ΔNLR = NLR_2–8 weeks_ – NLR_baseline_ ≥ 1) is also associated with TTD and OS with HRs of 2.6 (*p* = 2.2 × 10^− 7^, CI = 1.8–3.8) and 3.3 (*p* = 8.4 × 10^− 8^, CI = 2.1–5.2), respectively (Fig. 2d and h represent the corresponding survival curves). While high NLR at baseline is associated with shorter OS (Fig. 2e), the impact on OS in the NLR ≥ 5 patient group is further manifested with sustained high NLRs over the course of treatment (Fig. 2f and g). Supplementary Figure 1 depicts the median values of the NLR over all individuals for the 5 response groups: response, stable disease, progressive disease, putative HPD (hyper-progressive disease) and unknown during the 14 weeks after first ICI administration using a sliding window approach with 2 week intervals. A large increase in the NLR was notably strong in those with putative HPD, whereas those with response or stable disease tending to have stable levels of NLR.

For those with tumor response information, we examined overall response rate defined here as either complete or partial response (*n* = 65) or non-response (stable disease or progression, *n* = 101) according to physicians’ notes. Baseline NLR was not associated with response rate in our study (OR = 0.90, Table 1A), while those with a high NLR ≥ 5 at 2–8 weeks and 8–14 weeks are less likely to be in the response group than those with a normal NLR though the result was not statistically significant (Table 1B and C, ORs of 0.75 and 0.69, respectively). An increase in the NLR between baseline and 2–8 weeks or 8–14 weeks showed modestly significant correlation with lack of response (Table 1D and E) with ORs of 0.47 and 0.29 (*p*-values of 0.069 and 0.00096), respectively. Similar results were found if the analyses are restricted to individuals not on ICI/chemotherapy combinations, suggesting this result is not being driven by the higher response rate in the ICI/chemotherapy combination group (data not shown).

**Table 1:** Correlation between NLR and radiographic response

	Response	Non-response	Unknown	Response Rate	Odds Ratio (*p* value)
**A. Baseline NLR**					
NLR < 5	41	60	45	41%	0.90 (*p* = 0.87)
NLR ≥ 5	24	39	36	38%	
**B. 2–8 weeks NLR**					
NLR < 5	44	58	31	43%	0.75 (*p* = 0.41)
NLR ≥ 5	21	37	38	36%	
**C. 8–14 weeks NLR**					
NLR < 5	40	49	18	45%	0.69 (*p* = 0.31)
NLR ≥ 5	22	39	9	36%	
**D. ΔNLR between baseline and 2–8 weeks**					
ΔNLR< 1	53	64	37	45%	0.47 (*p* = 0.069)
ΔNLR≥1	12	31	32	28%	
**E. ΔNLR between baseline and 8–14 weeks**					
ΔNLR< 1	50	48	16	51%	0.29 (*p* = 0.00096)
ΔNLR≥1	12	40	11	23%	

*OR* odds ratio (for response in the NLR ≥ 5 group vs. the NLR < 5 group or between the ΔNLR≥1 and ΔNLR< 1)

### Many common laboratory tests correlate with clinical outcomes in ICI-treated aNSCLC

Next, we investigated in addition to NLR if other common laboratory test results are also associated with clinical response and survival (Supplementary Results; Supplementary Table 2, 3, 4 and 5).

**Fig. 3:**
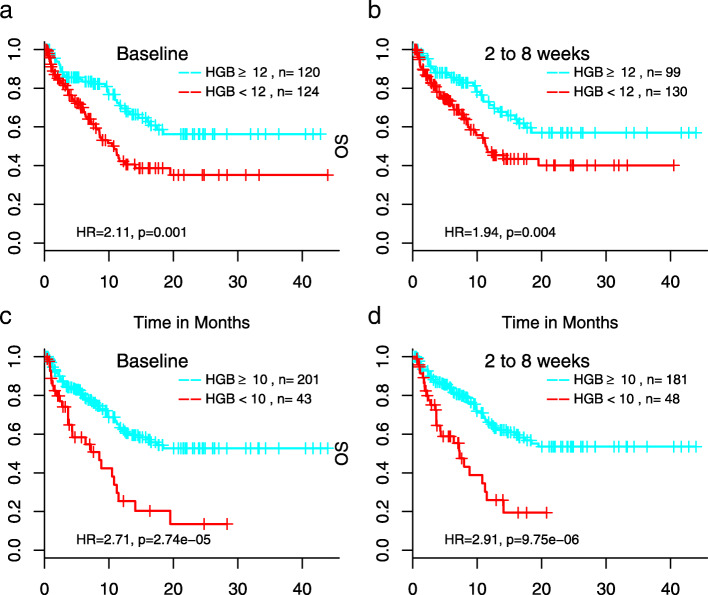
Association of hemoglobin with OS at baseline or 2–8 weeks after initiation of treatment. Cutoff of hemoglobin level to define anemia was 12 g/dL (**a** and **b**), the lower bound of reference range, or 10 g/dL (**c** and **d**), the definition of grade 2 or above adverse events according to NCI’s Common Terminology Criteria for Adverse Events (CTCAE)

Of most interest was HGB, the only test that at baseline correlated with both OS and response rate, where those with low baseline levels of HGB (< 12 g/dL) were less likely to be in the response group (OR = 0.46, *p*-value = 0.02; Supplementary Table 5). Though the association with response is modest, baseline HGB is associated with OS (HR = 2.11, p-value = 0.001). Low HGB, red blood cell (RBC) counts, and hematocrit (HCT), all signs of anemia and highly correlated with each other, were associated with shorter OS, with hazards that remain relatively constant over the time frame examined (Fig. 3, Supplementary Figure 2).

### Anemia correlates with response to ICI independently of NLR

In order to further stratify patient populations, we searched for variables that affect neutrophil levels. Identifying such variables does not only help to identify potential confounding factors that may bias results or create noise for association of NLR with outcome, but more importantly may aid in the identification of variables that are associated with outcome but not with neutrophil levels (and consequently not NLR), therefore representing independent factors for association with outcomes. Here we use absolute neutrophil count (ANC) rather than NLR as the NLR is a ratio and can create large outliers when lymphocyte counts are low.

We found many variables associated with ANC, including mutational status of EGFR/ALK, concurrent chemotherapy, concurrent infection, high level of troponin or other inflammatory markers, baseline levels or changes in electrolytes, and changes in lab tests associated with kidney or liver function (Supplementary Table 6). Concurrent chemotherapy and baseline inflammation were the only variables that decreased neutrophils. As expected, we see that individuals on concurrent chemotherapy experienced a decrease in the neutrophil levels as cytotoxic agents often destroy white blood cells (β = − 4.04, 1.00 × 10^− 05^). We also performed the analysis using a multivariable model, including variables with *p*-values< 0.05. Notably, baseline anemia and changes in anemia status were not associated with neutrophils. Thus, since baseline HGB correlates with response and survival (Fig. 3), HGB is likely a prognosticator of outcome independent of NLR.

### Developing a composite patient stratification marker of NLR and anemia for response to ICI

**Fig. 4:**
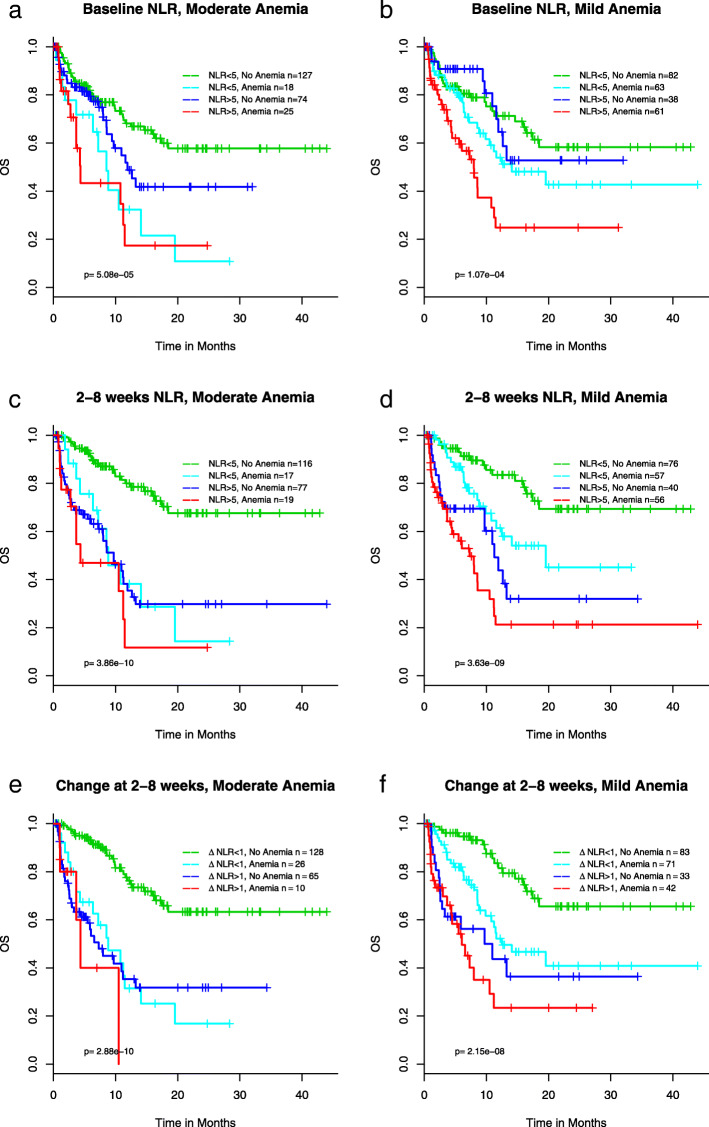
Association of a composite biomarker of NLR and hemoglobin and OS at baseline (**a**, **b**), 2–8 weeks (**c**, **d**), change of NLR from baseline to 2–8 weeks (**e**, **f**)

Since both NLR and anemia status correlate with clinical outcomes (Fig. 2 and Fig. 3), and anemia status is not associated with neutrophil levels as described above, we rationalized that a novel composite biomarker could be developed by combining NLR and anemia status. Figure 4 shows the KM curves for OS for the 4 groups: NLR < 5 + no anemia, NLR < 5 + anemia, NLR ≥ 5 + no anemia, NLR ≥ 5 + anemia for both mild and moderate anemia. Mild anemia was defined as HGB < 12 g/dL, the lower bound in the reference range (Supplementary Table 2), and moderate anemia was defined as HGB < 10 g/dL based on CTCAE (Common Terminology Criteria for Adverse Events) for grade 2 or above anemia published by National Cancer Institute (https://ctep.cancer.gov/protocoldevelopment/electronic_applications/docs/CTCAE_v5_Quick_Reference_8.5x11.pdf). Those with no anemia and NLR < 5 have significantly longer OS than both mild and moderate anemia, at baseline (Fig. 4a and b) and 2–8 weeks (Fig. 4c and d), and for ΔNLR> 1 (Fig. 4e and f). Notably, patients with NLR ≥ 5 and mild anemia prior to ICI therapy had the worst survival when compared to other patient groups (Fig. 4b); the median OS was 8.0 month (95CI: 4.5–11.5) in the NLR ≥ 5 and mild anemia group and not reached (95% CI 15.9-NE) in the rest of patients (*p* = 1.8 × 10^− 5^), hazard ratio 2.6 (95CI 1.7–4.1, *p* = 3.5 × 10^− 5^). When patients had pretreatment moderate anemia meeting the definition of adverse events in NCI’s CTCAE, they had poor survival regardless of NLR (Fig. 4a).

**Fig. 5:**
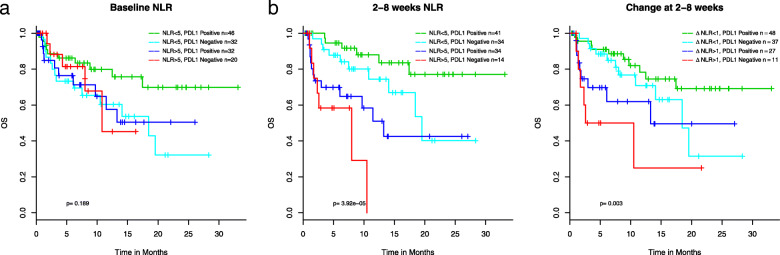
Association of combined PD-L1 status and NLR at baseline (**a**), and 2–8 weeks after treatment start (**b**) with OS

As PD-L1 expression status is a commonly used biomarker in aNSCLC for ICI therapy, we tested whether PD-L1 status, patients positive for the IHC staining proportion (1–100%) versus those with negative staining, were correlated with ANC and found no association (Supplementary Table 6). We then examined if the combination of NLR and PD-L1 status would help to stratify patients and identify a subpopulation who would achieve enhanced benefit from ICI treatment. As almost half of the patients in our cohort did not have PD-L1 tested, statistical power was significantly reduced for this analysis. Nevertheless, we observed a trend of longer survival in patients with baseline NLR < 5 and tumors positive for PD-L1, with the 3-year survival rate > 70% (Fig. 5a). However, NLR ≥ 5 at baseline (Fig. 5a) or at 2–8 weeks after the initiation of ICI (Fig. 5b) is associated with poor survival, regardless of PD-L1 status. We did not have a large enough sample to consider all three variables together (NLR, PD-L1 status, and anemia). Supplementary Table 7 further examines these predictors within both a good and a poor prognosis group (see Supplementary Results).

## Discussion

In this study, we confirmed previously reported association between high NLR and poor clinical outcomes in ICI-treated aNSCLC by elucidating such correlations at baseline as well as during the treatment. To expand on published findings, we further demonstrated the differences of survival in the high vs. low NLR patient populations during treatment is more profound than those at baseline (Fig. 2). Moreover, we show high NLR at baseline and to a lesser extent at 2–8 weeks negates the positive association between PD-L1 positivity and ICI response (Fig. 5a, b). To our knowledge, this has not been previously described in the literature and has clinical implications in that a sustained high level of NLR is particularly detrimental to patient outcomes and it is important to adequately manage patients’ blood counts during the course of ICI treatment.

We showed that results of many routine lab tests also correlate with clinical outcomes in our cohort. Of most interest are HGB, RBC counts and HCT where low levels at both baseline and during the treatment reflecting anemia were associated with shorter OS. The association between anemia and poor clinical outcomes in ICI-treated aNSCLC observed in this study has meaningful clinical implications. In the randomized phase 3 Keynote-189 [1] and IMpower-130 [3] studies evaluating pembrolizumab and atezolizumab, respectively, in combination with chemotherapy for the treatment of aNSCLC in the 1st line setting, similar percentage of patients experienced any grade anemia in the combination arm vs. the chemotherapy alone arm. However, in Checkmate-227 [2] study evaluating combination of two ICIs, nivolumab and ipilimumab for the front line treatment of aNSCLC, only 3.8% of patients in the nivolumab/ipilumumab arm had any grade anemia vs. 33% in the chemotherapy arm [2]. Therefore, our data suggest for those patients with low baseline HGB, RBC, and/or HCT levels, nivolumab/ipilimumab combination might be a more appropriate regimen than ICI-chemotherapy combinations.

Although we analyzed and reported results from data derived at both baseline and during treatment, the baseline results likely have more impact on clinical decision-making for selecting treatment options. As highlighted above, although baseline NLR does correlate with survival, the statistical significance is minimal. Furthermore, baseline NLR is not associated with response rate (Table 1). Therefore, a primary objective of this study was to develop composite baseline biomarkers by combining NLR and other variables that independently correlate with clinical outcomes. We were able to combine NLR and HGB to further stratify patient populations. We showed that patients with NLR ≥ 5 and mild anemia prior to ICI therapy had the worse survival when compared to other patient groups, and when patients had pretreatment anemia meeting the criteria in NCI’s CTCAE for grade 2 or above, they had poor survival regardless of NLR. Therefore, in the context of clinical practice, managing anemia to elevate patients’ HGB level prior to initiating ICI-based therapy may have clinical benefit and warrants further investigation. Alternatively, applying a non-chemotherapy containing regimen may help to alleviate anemia and improve clinical outcomes. We would like to point out that although the patients in this study received ICI-based therapy in different settings of line of therapy, when we only analyzed those patients treated with ICI as the 1st line therapy, the results were similar to those derived from the entire cohort of 249 patients. Therefore, the results from this retrospective study is clinical meaningful in the current clinical setting where ICI-chemo combinations or nivolumab/ipilimumab combination are the standard-of-care 1st line therapy in advanced NSCLC.

Recently, there are several published studies where composite biomarkers were developed for ICI response [17, 26, 32–34]. While these efforts combined multiple variables into a single numerical score to improve statistical associations with clinical endpoints, their clinical utility is limited largely due to the complexity of the scoring systems. Our composite biomarker of NLR and HGB are based on simple, well established cutoffs, and therefore, can be more easily adopted in clinical practice. Moreover, the composite marker of NLR and HGB is based on CBC tests that are routinely performed in all clinics, and could be particularly useful in certain countries and regions where PD-L1 testing may not be readily available.

We recognize there are significant limitations in this study. First, NLR, anemia, or the composite biomarker of NLR and HGB might be prognostic rather than predictive of ICI response. This type of biomarkers would not help us to understand the underlying mechanisms of innate or acquired resistance to ICI-based therapy to develop novel therapeutics or strategies for combination therapies. Second, the real-world data such as the cohort we analyzed in this study are intrinsically noisy. The medical records span many years with evolving treatment guidelines. The patients are heterogeneous in terms of therapies received and laboratory testing performed. In this 249-patient cohort, some patients received ICI-based therapy as the front line therapy, while others did as the 2nd, 3rd, or even later lines of therapy after disease progression on platinum-containing chemotherapy. Although we applied rigorous statistical methods to harmonize the data and to adjust for variables that may impact the results, the retrospective nature of the work requires replication in other cohorts. Of note, there are 45 patients who received ICI-chemotherapy combination as the 1st line therapy. When we removed these 45 patients from the cohort, correlation between NLR, HGB or the NLR-HGB composite marker with survival still remain (Supplementary Figures 3, and 4). Third, due to data limitations, our determination of HPD was based on TTF < 2 months, which is only an approximation of HPD defined by accelerated tumor growth rate or tumor growth kinetics upon anti-PD1/PD-L1 therapy. Finally, even we began with 11,138 NSCLC patients in MSHS, there were only 249 patients received ICI therapy by the cutoff date. This small sample size further emphasizes the need for additional validation.

## Conclusion

We developed a composite biomarker of NLR and HGB that could be applicable in the clinical decision-making process to optimize ICI-based therapy in aNSCLC. Our results warrant further investigations in larger patient populations and prospective clinical studies.

## Supplementary Information

**Additional file 1: Supplementary Fig. 1.** Median NLR over time by response group using a sliding window approach in 2 week intervals post treatment. For example, week 3 represents the median value for lab readings between 2 and 4 weeks over all individuals within the respective group, with the point size proportional to the number of individuals with available data in that interval. Best fit line is plotted for 0–10 weeks.

**Additional file 2: Supplementary Fig. 2.** Association of red blood cell counts (A and B), or hematocrit (C and D) with OS at baseline or 2–8 weeks after initiation of treatment.

**Additional file 3: Supplementary Fig. 3.** Association of NLR (A-D) or HGB (E-H) with OS in patients treated with ICI without concurrent chemotherapy. For NLR, Kaplan-Meier curves are shown for NLR ≥ 5 and NLR < 5 patients at baseline (A), 2–8 weeks (B), and 8–14 weeks (C), or increase in NLR from baseline to 2–8 weeks ≥1 and < 1 (D). For HGB, cutoff of hemoglobin level to define anemia was 12 g/dL (E and F), or 10 g/dL (G and H).

**Additional file 4: Supplementary Fig. 4.** Association of a composite biomarker of NLR and hemoglobin and OS at baseline in patients treated with ICI without concurrent chemotherapy.

**Additional file 5: Supplementary Table 1.** Published studies on correlations between NLR and clinical outcomes in ICI-treated aNSCLC.

**Additional file 6: Supplementary Table 2.** Reference range, names, and units of lab tests analyzed in this study. **Supplementary Table 3.** Association of lab test results with OS. HRs and *p*-values are given for different time points. Below/Above represents the number of individuals with lab values below and above the given threshold column B (or the number above/below a 15% change for column L). Column B gives the direction of the abnormal and value for the abnormal lab test. NE = Not estimable. **Supplementary Table 4.** Association of lab test results with TTD. HRs and p-values are given for different time points. Below/Above represents the number of individuals with lab values below and above the given threshold column B (or the number above/below a 15% change for column L). Column B gives the direction of the abnormal and value for the abnormal lab test. NE = Not estimable. **Supplementary Table 5.** Association of lab test results with radiographic response. ORs and p-values are given for different time points. Below/Above represents the number of individuals with lab values below and above the given threshold column B (or the number above/below a 15% change for column L). Column B gives the direction of the abnormal and value for the abnormal lab test. NE = Not estimable. **Supplementary Table 7.** Association of potential predictors with outcome in good and poor prognosis groups.

**Additional file 7: Supplementary Table 6.** Effect of variables on change in neutrophils from baseline to 2–8 weeks. Significant p-values (*p* < 0.05) are in bold.

**Additional file 8:** Supplementary Methods and Supplementary Results.

## Data Availability

The data that support the findings of this study have been originated by Sema4. These de-identified data may be made available upon request and are subject to a license agreement with Sema4; interested researchers should contact corresponding authors.

## Abbreviations

ICI: Immune checkpoint inhibitor
NSCLC: Non-small cell lung cancer
OS: Overall survival
TTD: Time to treatment discontinuation
CBC: Complete blood count
CMP: Comprehensive metabolic panel
NLR: Neutrophil-lymphocyte ratio
HGB: Hemoglobin
