# Supplementary figures and images for: A composite biomarker of neutrophil-lymphocyte ratio and hemoglobin level correlates with clinical response to PD-1 and PD-L1 inhibitors in advanced non-small cell lung cancers
Source: BMC Cancer. 2021 Apr 21;21:441. doi: 10.1186/s12885-021-08194-9 (PMC8059160; doi:10.1186/s12885-021-08194-9)

# NLR

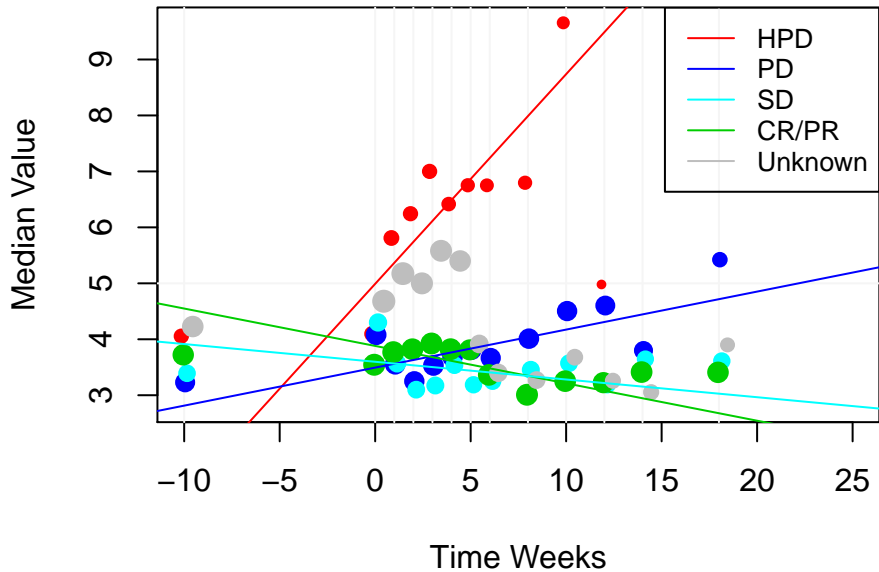

Supplement: Supplementary file 1 — Additional file 1: Supplementary Fig. 1. Median NLR over time by response group using a sliding window approach in 2 week intervals post treatment. For example, week 3 represents the median value for lab readings between 2 and 4 weeks over all individuals within the respective group, with the point size proportional to the number of individuals with available data in that interval. Best fit line is plotted for 0–10 weeks. [file 12885_2021_8194_MOESM1_ESM.pdf]

A

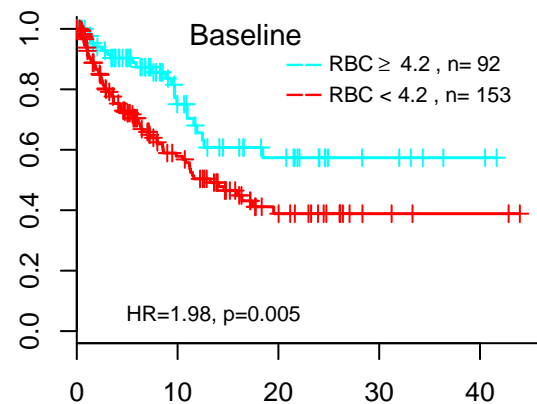

B

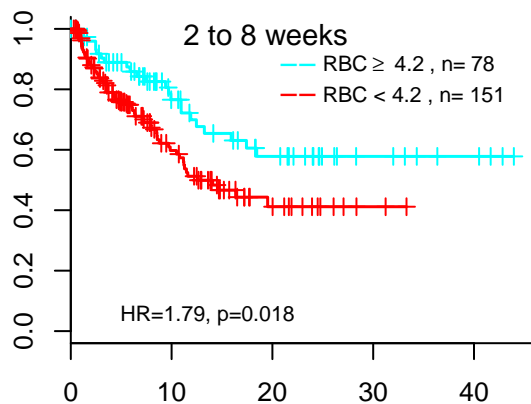

C

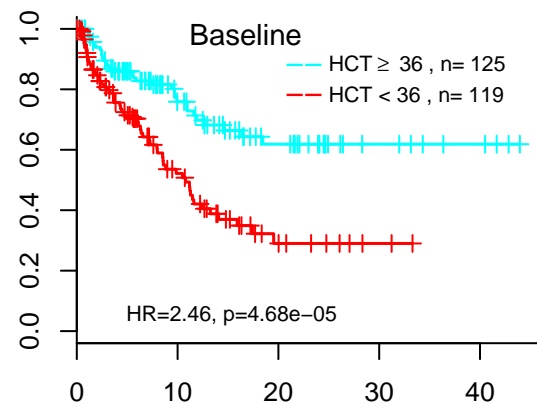

D

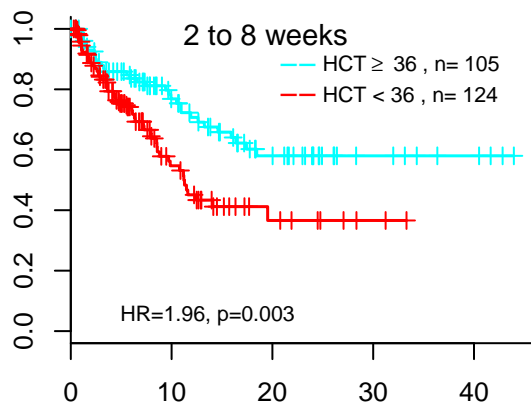

Supplement: Supplementary file 2 — Additional file 2: Supplementary Fig. 2. Association of red blood cell counts (A and B), or hematocrit (C and D) with OS at baseline or 2–8 weeks after initiation of treatment. [file 12885_2021_8194_MOESM2_ESM.pdf]

A

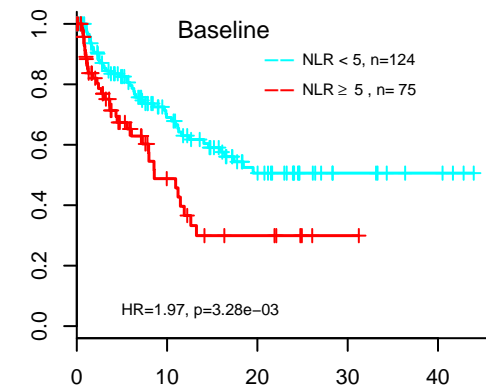

B

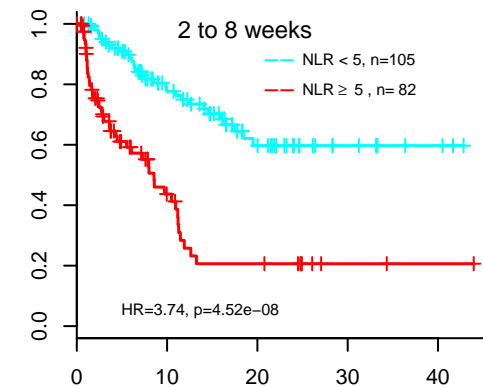

C

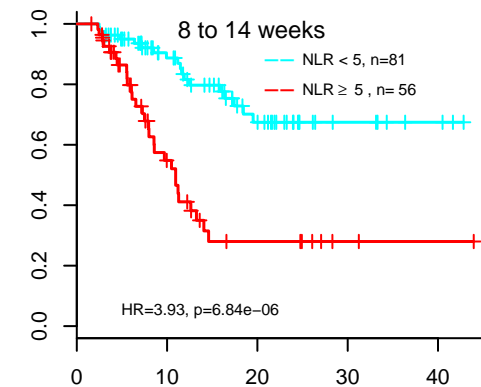

D

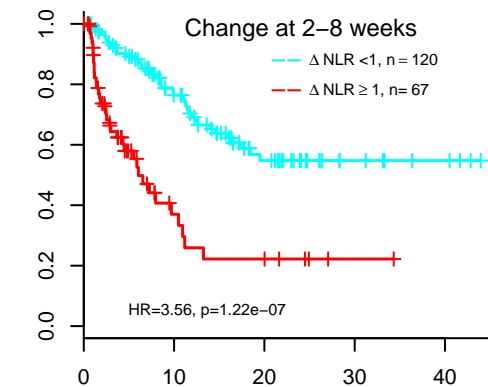

E

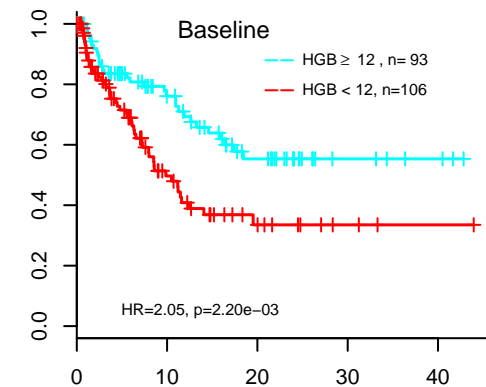

F

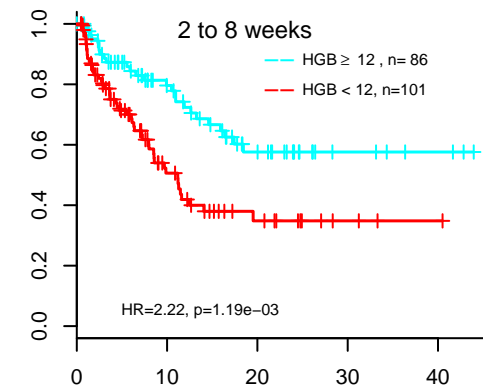

G

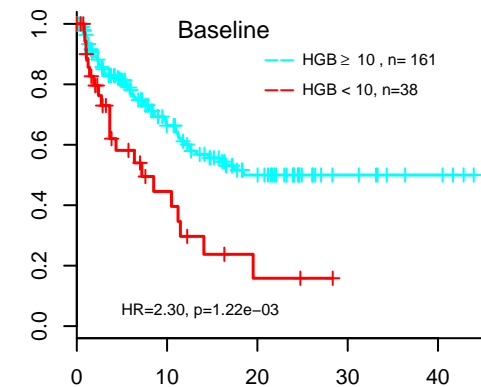

H

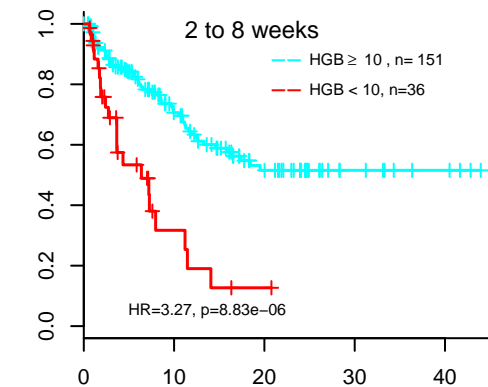

Supplement: Supplementary file 3 — Additional file 3: Supplementary Fig. 3. Association of NLR (A-D) or HGB (E-H) with OS in patients treated with ICI without concurrent chemotherapy. For NLR, Kaplan-Meier curves are shown for NLR ≥ 5 and NLR < 5 patients at baseline (A), 2–8 weeks (B), and 8–14 weeks (C), or increase in NLR from baseline to 2–8 weeks ≥1 and < 1 (D). For HGB, cutoff of hemoglobin level to define anemia was 12 g/dL (E and F), or 10 g/dL (G and H). [file 12885_2021_8194_MOESM3_ESM.pdf]

## Baseline Labs Without Concurrent Chemotherapy

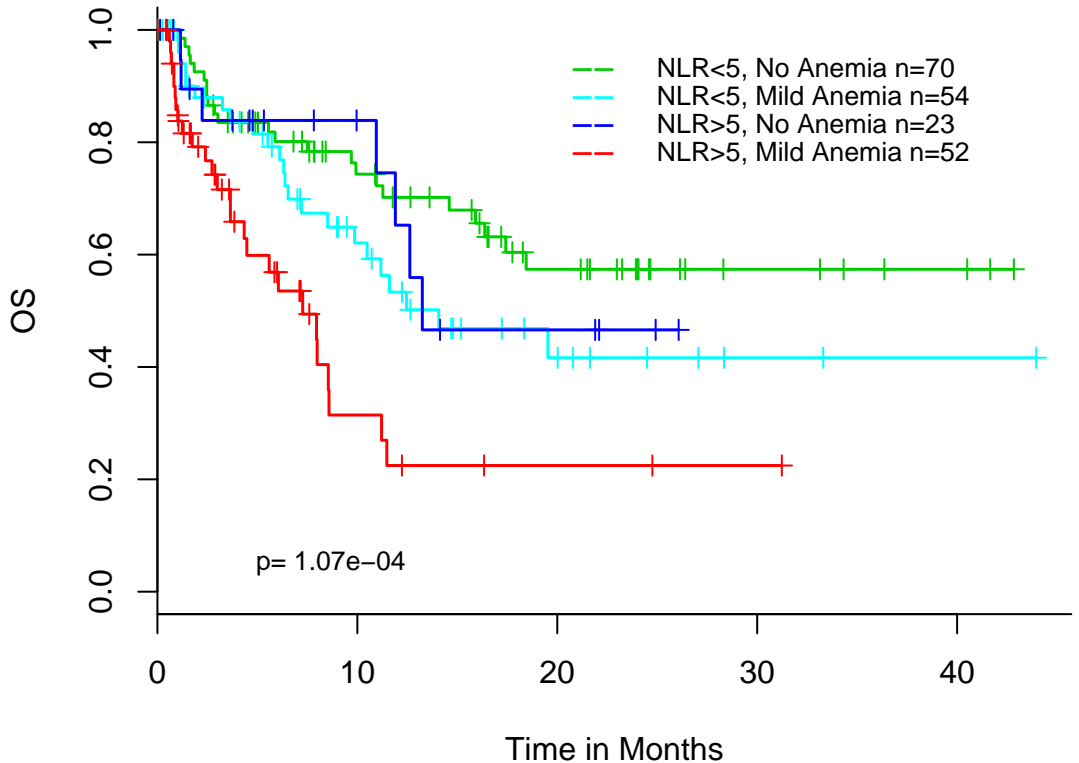

Supplement: Supplementary file 4 — Additional file 4: Supplementary Fig. 4. Association of a composite biomarker of NLR and hemoglobin and OS at baseline in patients treated with ICI without concurrent chemotherapy. [file 12885_2021_8194_MOESM4_ESM.pdf]
