# Supplementary material for: A composite biomarker of neutrophil-lymphocyte ratio and hemoglobin level correlates with clinical response to PD-1 and PD-L1 inhibitors in advanced non-small cell lung cancers
Source: BMC Cancer. 2021 Apr 21;21:441. doi: 10.1186/s12885-021-08194-9 (PMC8059160; doi:10.1186/s12885-021-08194-9)
Supplement: Supplementary file 5 — Additional file 5: Supplementary Table 1. Published studies on correlations between NLR and clinical outcomes in ICI-treated aNSCLC. [file 12885_2021_8194_MOESM5_ESM.docx]

Supplemental Table I

| Study | Variable | Threshold | Time | Outcome | n | HR | drugs |
| --- | --- | --- | --- | --- | --- | --- | --- |
| Kiriu et al 2017^1^ | ΔNLR | >30% change in NLR | after first cycle;  and second cycle | shorter TTF | 19 | p = 0.014;  p < 0.001 | nivolumab |
| Diem et al 2017^2^ | NLR | Elevated NLR | Pre-treatment NLR | worse OS;  lower response rates | 52 | 3.64, p<0.001;  0.17, p=0.013 | nivolumab |
| Takeda et al 2018^3^ | NLR | ≥5 | 4 weeks | Shorter PFS | 26 | 5.995 | nivolumab |
| Shiroyama et al 2018^4^ | NLR  (ECOG ≥2)  (albumin <3.7 g/dL) (ALI <18) | ≥4 | Pretreatment | Shorter PFS;  Early progression | 201 (Japan) | 1.46, p=0.02;  OR=2.28, p=0.005 |  |
| Shu et al 2018^5^ | NLR | ≥5 | 6 weeks | Shorter PRS  Shorter OS | 54 | 15.1, p < 0.001;  3.9, p=0.003 | nivolumab or  pembrolizumab |
| Fukui et al 2019 or 2018^6^ | NLR | ≥5 | Pretreatment | Poorer OS prognosis | 52 | 4.52, p=.013 | nivolumab |
| Zhuo et al 2018^7^ | Blood routine test |  | Post treatment | slight elevation of WBC; lymphocyte ratio was increased slightly in disease controlled patients but decreased prominently in disease progressed patients in response to therapy | 10 |  | atezolizumab |
| Park et al 2017^8^ | NLR & ΔNLR | NLR≥5& ΔNLR≥0 | Baseline + Change after first treatment | Shorter PFS | 159 | 3.12, p<.0001 | nivolumab |
| Mezquita et al 2018^9^ | neutrophils/(leukocytes minus neutrophils) ratio (dNLR) & LIPI score | dNLR≥3 | Baseline | Shorter OS  shorter PFS  non-DCR | 161 test set  (305 validation,  8 European centers) | 1.98, p=0.002;  1.61, p=0.01;  OR=2.12, p=0.03 | nivolumab, pembrolizumab, atezolizumab, durvalumab, and durvalumab-ipilimumab |
| Bagley et al 2017^10^ | NLR | NLR ≥ 5 | Pretreatment/Baseline | Shorter OS;  Shorter PFS | 175 | 2.07, p = 0.002;  1.43, p = 0.04 | nivolumab |
| Zer et al 2018^11^ | NLR, ANC (absolute neutrophil count) | NLR ≤4 and ANC | Baseline, week 2 or 3 and week 8 | Baseline NLR: superior DCR ; treatment duration  time to progression; OS;  Post treatment NLR & ANC: treatment response and duration | 88 |  | PD-1 axis inhibitors |
| Kazandijan et al 2019^12^ | prognostic index composite (LIPI) score (dNLR and LDH) | good LIPI score | Baseline | better OS;  better PFS; | 1368 (11 mNSCLC randomized trials) | 0.34; 95% CI, 0.28-0.42;  0.56; 95% CI, 0.45-0.68 | ICIs |
| Dusselier et al 2019^13^ | ΔNLR | ΔNLR < 1 | Between Baseline and 4^th^ treatment | Better OS | 59 | 0.29, 0.001 | nivolumab |
| Liu et al 2019^14^ | NLR;PLR;SII | NLR≤3.07 | Baseline (<7 days) | better PFS;  better OS | 44 Chinese | 0.46, p = 0.048;  0.20, p = 0.002 |  |
| Ruiz-Bañobre et al 2019^15^ | prognostic index composite (LIPI) score (dNLR and LDH) | Low LIPI score | Baseline (<30 days) | poorer OS;  poorer PFS;  lower DCR (disease control rate) | 188 multicenter | 3.12, p<0.0001;  1.45, p=0.03;  0.41, p=0.001 | nivolumab |
| Prelaj et al 2020^16^ | NLR, dNLR  change of NLR (Δ NLR) from baseline to second cycle | NLR ≥ 4  dNLR ≥ 2.2 | Baseline, after 1st cycle(3wks), after 2^nd^ cycle(6wks) | PFS | 154 | Δ NLR of >30% from baseline to the second cycle worse PFS (HR 1.52 p=0.04); NLR & dNLR sig poorer OS/PFS all cycles | pembrolizumab 2^nd^ line plus |
| Prelaj et al 2019^17^ | NLR | NLR ≥ 4 | Baseline | PFS | 193 (Italy) | 1.49, p = 0.029 | anti-PD-1/PD-L1 inhibitors |

**References**

1. Kiriu T, Yamamoto M, Nagano T, et al. The time-series behavior of neutrophil-to-lymphocyte ratio is useful as a predictive marker in non-small cell lung cancer. *Plos One*. 2018;13(2):e0193018. doi:10.1371/journal.pone.0193018

2. S D, S S, M K, et al. Neutrophil-to-Lymphocyte ratio (NLR) and Platelet-to-Lymphocyte ratio (PLR) as prognostic markers in patients with non-small cell lung cancer (NSCLC) treated with nivolumab. *Lung Cancer*. Published online 2017. doi:10.1016/j.lungcan.2017.07.024

3. Takeda T, Takeuchi M, Saitoh M, Takeda S. Neutrophil-to-lymphocyte ratio after four weeks of nivolumab administration as a predictive marker in patients with pretreated non-small-cell lung cancer. *Thorac Cancer*. 2018;9(10):1291-1299. doi:10.1111/1759-7714.12838

4. Shiroyama T, Suzuki H, Tamiya M, et al. Pretreatment advanced lung cancer inflammation index (ALI) for predicting early progression in nivolumab‐treated patients with advanced non–small cell lung cancer. *Cancer Med-us*. 2018;7(1):13-20. doi:10.1002/cam4.1234

5. Suh KJ, Kim SH, Kim YJ, et al. Post-treatment neutrophil-to-lymphocyte ratio at week 6 is prognostic in patients with advanced non-small cell lung cancers treated with anti-PD-1 antibody. *Cancer Immunol Immunother*. 2018;67(3):459-470. doi:10.1007/s00262-017-2092-x

6. Fukui T, Okuma Y, Nakahara Y, et al. Activity of Nivolumab and Utility of Neutrophil-to-Lymphocyte Ratio as A Predictive Biomarker for Advanced Non-Small Cell Lung Cancer: A Prospective Observational Study. *Clin Lung Cancer*. 2018;20(3):208-214.e2. doi:10.1016/j.cllc.2018.04.021

7. Zhuo M, Chen H, Zhang T, et al. The potential predictive value of circulating immune cell ratio and tumor marker in atezolizumab treated advanced non-small cell lung cancer patients. *Cancer Biomark*. 2018;22(3):467-476. doi:10.3233/cbm-171089

8. Park W, Kwon D, Saravia D, et al. Developing a Predictive Model for Clinical Outcomes of Advanced Non-Small Cell Lung Cancer Patients Treated With Nivolumab. *Clin Lung Cancer*. 2017;19(3):280-288.e4. doi:10.1016/j.cllc.2017.12.007

9. Mezquita L, Auclin E, Ferrara R, et al. Association of the Lung Immune Prognostic Index With Immune Checkpoint Inhibitor Outcomes in Patients With Advanced Non-Small Cell Lung Cancer. *JAMA Oncol*. Published online n.d. doi:10.1001/jamaoncol.2017.4771

10. Bagley SJ, Kothari S, Aggarwal C, et al. Pretreatment neutrophil-to-lymphocyte ratio as a marker of outcomes in nivolumab-treated patients with advanced non-small-cell lung cancer. *Lung Cancer*. 2017;106:1-7. doi:10.1016/j.lungcan.2017.01.013

11. Zer A, Sung MR, Walia P, et al. Correlation of Neutrophil to Lymphocyte Ratio and Absolute Neutrophil Count With Outcomes With PD-1 Axis Inhibitors in Patients With Advanced Non-Small-Cell Lung Cancer. *Clin Lung Cancer*. 2018;9(5):426-434.e1. doi:10.1016/j.cllc.2018.04.008

12. Kazandjian D, Gong Y, Keegan P, Pazdur R, Blumenthal GM. Prognostic Value of the Lung Immune Prognostic Index for Patients Treated for Metastatic Non–Small Cell Lung Cancer. *Jama Oncol*. 2019;5(10):1481. doi:10.1001/jamaoncol.2019.1747

13. Dusselier M, Deluche E, Delacourt N, et al. Neutrophil-to-lymphocyte ratio evolution is an independent predictor of early progression of second-line nivolumab-treated patients with advanced non-small-cell lung cancers. *Plos One*. 2019;14(7):e0219060. doi:10.1371/journal.pone.0219060

14. Liu J, Li S, Zhang S, et al. Systemic immune‐inflammation index, neutrophil‐to‐lymphocyte ratio, platelet‐to‐lymphocyte ratio can predict clinical outcomes in patients with metastatic non‐small‐cell lung cancer treated with nivolumab. *J Clin Lab Anal*. 2019;33(8). doi:10.1002/jcla.22964

15. Ruiz-Bañobre J, Areses-Manrique MC, Mosquera-Martínez J, et al. Evaluation of the lung immune prognostic index in advanced non-small cell lung cancer patients under nivolumab monotherapy. *Transl Lung Cancer Res*. 2019;8(6):1078-1085. doi:10.21037/tlcr.2019.11.07

16. Prelaj A, Rebuzzi SE, Pizzutilo P, et al. EPSILoN: A Prognostic Score Using Clinical and Blood Biomarkers in Advanced Non-Small-cell Lung Cancer Treated With Immunotherapy. *Clin Lung Cancer*. Published online 2020. doi:10.1016/j.cllc.2019.11.017

17. Prelaj A, Ferrara R, Rebuzzi SE, et al. EPSILoN: A Prognostic Score for Immunotherapy in Advanced Non-Small-Cell Lung Cancer: A Validation Cohort. *Cancers*. 2019;11(12):1954. doi:10.3390/cancers11121954
