# Supplementary material for: A composite biomarker of neutrophil-lymphocyte ratio and hemoglobin level correlates with clinical response to PD-1 and PD-L1 inhibitors in advanced non-small cell lung cancers
Source: BMC Cancer. 2021 Apr 21;21:441. doi: 10.1186/s12885-021-08194-9 (PMC8059160; doi:10.1186/s12885-021-08194-9)
Supplement: Supplementary file 7 — Additional file 7: Supplementary Table 6. Effect of variables on change in neutrophils from baseline to 2–8 weeks. Significant p-values (p < 0.05) are in bold. [file 12885_2021_8194_MOESM7_ESM.docx]

Supplementary Table 6.

|  |  | **Single Variable Model** | | | **Multivariable Model** | | |
| --- | --- | --- | --- | --- | --- | --- | --- |
| Variable | Category | n | β (effect size) | p-value | n | β (effect size) | p-value |
| *PD-L1 Status* | |  |  |  |  |  |  |
|  | Negative | 48 | Ref |  |  |  |  |
|  | Positive (1-100%) | 75 | 0.78 | 0.441 |  |  |  |
| *Target Mutation in EGFR/ALK* | |  |  |  |  |  |  |
|  | No | 147 | Ref |  | 146 | Ref |  |
|  | Yes | 22 | 3.42 | **0.006** | 22 | 1.99 | 0.063 |
| *Smoking Status* | |  |  |  |  |  |  |
|  | Never Smoker | 32 | Ref |  |  |  |  |
|  | Current or Former Smoker | 197 | -1.17 | 0.265 |  |  |  |
| *ECOG Score* | |  |  |  |  |  |  |
|  | High 2/3 | 35 | Ref |  |  |  |  |
|  | Low 0/1 | 122 | 1.52 | 0.152 |  |  |  |
|  | Missing | 72 | 0.89 | 0.437 |  |  |  |
| *Recent Chemotherapy* | |  |  |  |  |  |  |
|  | No | 191 | Ref |  |  |  |  |
|  | Within 30 days | 38 | 1.15 | 0.240 |  |  |  |
| *Concurrent Chemotherapy* | |  |  |  |  |  |  |
|  | No | 187 | Ref |  | 186 | Ref |  |
|  | Yes | 42 | -4.04 | **1.00x10^-05^** | 42 | -3.46 | **2.00x10^-05^** |
| *CSF RX for neutropenia* | |  |  |  |  |  |  |
|  | No | 216 | Ref |  |  |  |  |
|  | Yes | 13 | -0.69 | 0.661 |  |  |  |
| *Concurrent infection* | |  |  |  |  |  |  |
|  | No | 215 | Ref |  | 214 | Ref |  |
|  | Yes | 14 | 3.42 | **0.024** | 14 | 2.36 | 0.073 |
| *Post RX Rash* | |  |  |  |  |  |  |
|  | No | 198 | Ref |  |  |  |  |
|  | Yes | 31 | -0.61 | 0.568 |  |  |  |
| *High Troponin* | |  |  |  |  |  |  |
|  | No | 204 | Ref |  | 203 | Ref |  |
|  | Yes | 25 | 4.66 | **5.00x10^-05^** | 25 | 2.04 | 0.053 |
| *Electrolyte Score* | |  |  |  |  |  |  |
|  | Baseline | 229 | -2.01 | **3.30x10^-04^** | 228 | -0.72 | 0.167 |
|  | Δ 2-8 weeks | 229 | 2.47 | **<1.00x10^-05^** | 228 | 1.57 | **0.001** |
| *Mild Anemia: HGB<12* | |  |  |  |  |  |  |
|  | Baseline | 229 | 0.28 | 0.707 |  |  |  |
|  | Δ 2-8 weeks | 229 | -0.36 | 0.643 |  |  |  |
| *Liver Function* | |  |  |  |  |  |  |
|  | Baseline | 228 | -0.72 | 0.120 |  |  |  |
|  | Δ 2-8 weeks | 228 | 1.12 | **0.016** | 228 | 0.53 | 0.193 |
| *Kidney Function* | |  |  |  |  |  |  |
|  | Baseline | 229 | -0.02 | 0.975 |  |  |  |
|  | Δ 2-8 weeks | 229 | 2.79 | **8.00x10^-05^** | 228 | 2.04 | **0.001** |
| *Inflammation Score* | |  |  |  |  |  |  |
|  | Baseline | 229 | -3.67 | **0.001** | 228 | -3.78 | **5.00x10^-05^** |
|  | 2-8 weeks | 229 | 0.9 | 0.369 |  |  |  |
