# Supplementary material for: A composite biomarker of neutrophil-lymphocyte ratio and hemoglobin level correlates with clinical response to PD-1 and PD-L1 inhibitors in advanced non-small cell lung cancers
Source: BMC Cancer. 2021 Apr 21;21:441. doi: 10.1186/s12885-021-08194-9 (PMC8059160; doi:10.1186/s12885-021-08194-9)
Supplement: Supplementary file 8 — Additional file 8: Supplementary Methods and Supplementary Results. [file 12885_2021_8194_MOESM8_ESM.docx]

# **A composite biomarker of neutrophil-lymphocyte ratio and hemoglobin level correlates with clinical response to PD-1 and PD-L1 inhibitors in advanced non-small cell lung cancers**

Kristin L. Ayers^1^, Meng Ma^1^, Gaspard Debussche^1^, David Corrigan^1^, Jonathan McCafferty^1^, Kyeryoung Lee^1^, Scott Newman^1^, Xiang Zhou^1^, Fred R. Hirsch^2^, Philip C. Mack^2^, Jane J. Liu^1,3^, Eric E. Schadt^1,2*^, Rong Chen^1,2*^, Shuyu D. Li^1,2*^

^1^Sema4, a Mount Sinai Venture, 333 Ludlow Street, Stamford, CT 06902, USA

^2^Center of Thoracic Oncology/Tisch Cancer Institute and Icahn School of Medicine, Mount Sinai, 1 Gustave L. Levy Pl, New York, NY 10029, USA

^3^Illinois CancerCare, 8940 N Wood Sage Rd, Peoria, IL 61615

^*^Corresponding Authors:

Eric E. Schadt, a Mount Sinai Venture, 333 Ludlow Street, Stamford, CT 06902

([eric.schadt@sema4.com](mailto:eric.schadt@sema4.com))

Rong Chen, Sema4, a Mount Sinai Venture, 333 Ludlow Street, Stamford, CT 06902 ([rong.chen@sema4.com](mailto:rong.chen@sema4.com))

Shuyu D. Li, Sema4, a Mount Sinai Venture, 333 Ludlow Street, Stamford, CT 06902 ([shuyudanli@gmail.com](mailto:shuyudanli@gmail.com))

**Supplementary Methods**

*Blood Counts and other lab tests*

The results of all lab tests were extracted from the EHR. The time in days of the test was computed as the difference from the first administration of immunotherapy to the lab test dates. Time could be negative if tests were performed before treatment. All tests from the CBC panel and the CMP that had less than 3% missing data at baseline and less than 10% missing data at the 2-8 weeks interval were included in the analyses. Some tests with higher percentage of missing data that were previously associated with cancer outcome in the literature, were also considered for evaluation (e.g., Lactate dehydrogenase;LDH). For lab tests where both high and low readings are considered abnormal and the test did not appear to be previously associated with cancer outcome in a search of the literature, the direction for an abnormal reading was determined as the direction with the larger number of individuals outside of the range for the current cohort. For changes in lab values between time points, individuals were categorized by presence or absence of more than 15% change in levels between the two time points relative to the width of the reference range (Supplementary Table 2). For example, for neutrophils where high levels were considered abnormal, the threshold would be: (NEU_2-8 weeks_ -NEU_baseline_)/(7.0-1.7)> 0.15, where 1.7 and 7.0 in the denominator represent the low and high thresholds, respectively, for the neutrophil reference ranges given in Supplementary Table 2.

*Chemotherapy and Neutropenia drugs*

Neutropenia generally occurs 7-12 days after chemotherapy administration and continues for several days [<https://www.cdc.gov/cancer/preventinfections/pdf/neutropenia.pdf>]. White blood cell growth factors also known as hematopoietic colony-stimulating factors (CSFs) are drugs that are given during chemotherapy to mitigate the risk of infection by enabling the body to make more WBCs to prevent neutropenia. Drugs include filgrastim, pegfilgrastim, and sargramostim and are generally given a day after chemotherapy treatment. Because these chemotherapy and CSFs can temporarily affect neutrophil levels, it is important to consider their potential confounding effects on outcomes. For example, if individuals with poor performance status are more likely to take these drugs, then high neutrophils could be related to overall status rather than immunotherapy treatment effects. To find these individuals we extracted all chemotherapy and CSF treatments from medications given in the EHR. For CSF, we did a string search for any drug name matching filgrastim, neupogen, pegfilgrastim, neulasta, lenograstim, granocyte, sargramostim, leucine, molgramostim or leucomax. We recorded individuals who were given CSF at the first ICI dose, 0-8 weeks after the first dose, or 8-14 weeks after the first ICI dose. All chemotherapy medications given were also extracted and it was determined when an individual had no record of chemotherapy, had ICI after chemotherapy, had concurrent chemotherapy with ICI, or started chemotherapy after ICI began. We identified 76 individuals in which CSF treatment appeared in their records, with 40 of these individuals having CSF treatment occurring prior to the start of the IO (all 40 of these patients where on chemotherapy before IO). Twenty-one patients were given CSFs between 0-14 weeks after IO began, of which 15 were being treated with chemotherapy concurrently at ICI start, but CSFs did not appear to be administered until after ICI treatment began.

*Infection*

Neutrophil and other blood cell levels can change in response to infection and thus confounding should be considered between infection, the NLR, and outcome. We extracted individuals with ICD9/10 codes that included ‘bronch’,’infection’, ‘bacter’ and ‘pneumo’ and filtered appropriately after a manual review of terms. For our purposes we are primarily investigating whether infection may be correlated with the changes in neutrophil levels during the course of treatment. As lung infections are common in lung cancer, infections were grouped into pneumonia, bronchitis, other lung infection, and non-lung local infection (such as infection at catheter site). Timings for the infection were noted and we consider those with an ICD code for infection between 30 days before and up to 8 weeks after the 1^st^ ICI administration as having a concurrent infection.

*Rash*

Skin conditions are thought to be correlated with response to ICI treatment. ICD codes for skin diagnoses were extracted from the EMR for rash, pruritus, bulla, and psoriasis including the ICD9 and ICD10 codes: 782.1, 698.9, 694.9, 696.1, R21, L29.9, L13.9 and L40.0 and were manually reviewed to determine the cause of rash. An individual was considered to have a post-treatment rash if the rash was diagnosed within one year of treatment and thought to be due to ICI treatment, and the individual was still on treatment at diagnosis. For this reason, rash is not a true predictive variable, but was included to examine correlations with other variables.

*Composite lab scores*

As many lab tests are highly correlated and impact overall prognosis, we grouped test results by effects to create simple composite scores for liver function, kidney function, anemia, electrolyte imbalances, and inflammation. Our strategy was to create a simple sum assuming that the more abnormal lab tests an individual has, the poorer the condition of the patient (without double counting for highly correlated tests). These scores were not meant to serve as a predictive test, but rather an attempt to test organ systems for correlation with other variables associated with outcome. Scores for each group were computed for baseline levels and at 2-8 weeks after treatment. Each individual was given 1 point for each of the labs that fell above or below the following thresholds: (1) Electrolyte imbalance= Sodium <135 + Calcium >= 10.2 + Chloride <98 + Potassium >=5.5 + Magnesium <1.5; (2) Liver= ALP >147 + Albumin <3.5 + Bilirubin >1 + (Alanine aminotransferase (ALT) > 40 or Aspartate aminotransferase (AST) > 40); 3) Anemia as Moderate Anemia: HGB<10 or Mild Anemia: HGB<12; 4) Inflammation=CRP >10 + LDH >333; and 5) Kidney= Blood urea nitrogen(BUN) >22 + Creatinine >1.21. Those who were missing values at 2-8 weeks were assigned their baseline levels. For less often performed tests like LDH and CRP individuals were assigned 0 points for no test or normal values and 1 point for abnormal values. These scores were assessed for correlation with a change in neutrophil levels between baseline and 2-8 weeks. Additionally, an overall prognostic score was developed by summing these 5 scores. We chose the threshold that gave the most significant difference between the 2 groups: Poor prognosis with a score >1, and good prognosis with a score≤1 in order to examine variables within these groups.

**Supplementary Results**

*Correlation of common laboratory tests with clinical outcomes in ICI-treated aNSCLC*

The analyzed lab tests, abbreviations, descriptions, and reference ranges are listed in Supplementary Table 2. Supplementary Tables 3 and 4 provide hazard ratios and p-values for OS and TTD respectively for baseline, 2-8 weeks, 8-14 weeks, and the change between baseline and 2-8 weeks. In Supplementary Table 3, most of the blood counts and results from many other lab tests show modest association with OS at baseline as well as during treatment. Association with TTD (Supplementary Table 4) and the documented clinical response (Supplementary Table 5) was only observed during the treatment at 2-8 weeks and 8-14 weeks, with the exception that baseline hemoglobin (HGB) level correlate with radiographic responses. These lab tests may detect side effects of ICI treatment, side effects of chemotherapy, or potentially the extent of metastasis. LDH, which has previously been reported, had a modest impact on TTD and OS in our cohort, though the data for LDH is missing in many patients. We found alkaline phosphatase (ALP) levels >147 IU/L to be associated with OS at all time points. A recent study found colorectal cancer patients with high ALP levels have a 5.5 times greater odds of having a liver metastasis compared to those with normal ALP levels, and liver metastasis is known to be associated with poorer cancer outcome.

*Correlation of NLR with prognosis groups*

We assessed the effect of NLR in the poor and good prognosis groups defined by utilizing 5 categories of common laboratory tests reflecting anemia, liver function, kidney function, immune function and other general physiological functions of human body (see Supplementary Methods). In Supplementary Table 7 we examine PD-L1 status, baseline NLR, ΔNLR, baseline HGB, and rash as predictors of outcome in both the good and poor prognosis group. ΔNLR≥1 is significantly associated with lower TTD and OS in both prognosis groups. While not statistically significant, it is also correlated with lack of response (OR=0.51for the poor prognosis group and OR=0.38 for the good prognosis group). PD-L1 status is modestly associated with OS in both groups, and is associated with response, especially in the poor prognosis group (OR=6.22, p-value=0.009).
